# Supplementary material for: Transcriptome Analysis and QTL Mapping Identify Candidate Genes and Regulatory Mechanisms Related to Low-Temperature Germination Ability in Maize
Source: Genes (Basel). 2023 Oct 8;14(10):1917. doi: 10.3390/genes14101917 (PMC10606144; doi:10.3390/genes14101917)
Supplement: Supplementary file 1 [file genes-14-01917-s001.zip › genes-2629974-supplementary.pdf]

Table S1 Base data of RNA-sequencing

Sample	Clean reads pairs	Clean base(bp)	GC(%)	Q30(%)
Y0d-1	23,162,407	6,796,656,130	51.9; 52.9	95.9; 94.6
Y0d-2	25,634,775	7,402,676,223	53.6; 53.4	95.5; 94.5
Y0d-3	24,033,695	7,027,888,250	51.2; 52.2	95.9; 94.5
Y1d-1	34,146,056	10,033,225,001	51.8; 53.1	96.1; 94.9
Y1d-2	20,804,649	6,097,329,871	51.7; 52.9	96.0; 94.3
Y1d-3	29,270,351	8,572,658,563	52.3; 53.4	96.07; 93.9
Y3d-1	40,664,251	11,903,399,025	52.9; 54.1	96.0; 94.43
Y3d-2	31,614,601	9,230,476,276	52.5; 54.0	96.2; 90.8
Y3d-3	21,672,384	6,323,775,929	52.8; 54.1	96.1; 94.0
Y5d-1	22,208,096	6,451,057,429	53.2; 54.3	96.1; 94.2
Y5d-2	33,340,444	9,781,116,847	53.1; 54.2	96.1; 93.9
Y5d-3	39,317,896	11,501,685,020	52.4; 53.5	96.0; 93.9
Q0d-1	28,868,388	8,430,927,446	52.0; 53.1	98.9; 98.4
Q0d-2	22,243,431	6,484,097,726	52.4; 53.6	98.8; 98.3
Q0d-3	34,396,266	10,041,148,107	52.3; 53.4	98.9; 98.2
Q1d-1	36,013,195	10,533,646,257	52.5; 53.7	98.9; 98.3
Q1d-2	26,188,695	7,675,333,283	53.2; 54.4	98.9; 98.4
Q1d-3	23,056,474	6,741,737,931	52.9; 54.1	98.9; 98.3
Q3d-1	32,918,995	9,619,442,609	53.0; 54.2	98.9; 98.1
Q3d-2	39,497,155	11,564,230,368	54.1; 55.4	98.9; 98.1
Q3d-3	24,658,625	7,231,915,748	54.1; 55.4	98.9; 98.1
Q5d-1	23,610,847	6,925,408,007	53.1; 54.5	98.9; 98.1
Q5d-2	23,516,580	6,882,567,024	53.2; 54.4	98.9; 98.4
Q5d-3	25,895,402	7,592,220,745	52.5; 53.7	98.9; 98.2
Total	686,733,658	200,844,619,815	–	–
Mean	28,613,902	8,368,525,826	52.69; 53.85	97.4; 96.1

Table S2 Expression level of candidate genes in two genotypes at low-temperature germination stages

GeneID	T0d	T1d	T3d	T5d	Y0d	Y1d	Y3d	Y5d
Zm00001d031655	3.6	4.1	6.5	9.7	3.1	3.7	7.6	14.9
Zm00001d031951	1.7	3.0	3.5	5.6	0.7	3.7	6.8	16.0
Zm00001d031953	0.0	0.0	0.0	0.0	0.0	1.4	1.6	7.3
Zm00001d031429	435.5	552.6	446.0	396.8	490.4	774.5	790.7	571.2
Zm00001d031560	140.3	160.2	129.8	130.8	121.3	241.3	224.8	169.7
Zm00001d031640	245.6	338.6	272.2	230.4	226.1	477.4	425.0	259.0
Zm00001d031651	63.9	62.6	64.7	56.6	86.8	133.0	147.4	106.4
Zm00001d031667	8.5	9.6	9.2	13.2	3.0	5.0	4.5	6.3
Zm00001d031617	47.6	48.8	50.4	60.9	18.8	22.5	35.8	40.0
Zm00001d031992	5.1	5.8	6.1	7.6	1.3	2.4	3.2	3.3

Table S3 The significantly enriched KEGG pathways of the target modules

ID	Description	*q*-value
MEbrown：		
ID	Description	qvalue
zma00402	Benzoxazinoid biosynthesis	1.54E-05
zma00940	Phenylpropanoid biosynthesis	6.46E-03
zma04814	Motor proteins	6.46E-03
zma04075	Plant hormone signal transduction	6.46E-03
zma00280	Valine, leucine and isoleucine degradation	6.46E-03
zma00999	Biosynthesis of various plant secondary metabolites	6.46E-03
zma00650	Butanoate metabolism	6.55E-03
zma00250	Alanine, aspartate and glutamate metabolism	6.73E-03
zma00270	Cysteine and methionine metabolism	2.32E-02
MEred：		
zma03010	Ribosome	6.31E-132
zma03030	DNA replication	1.47E-13
MEgreen:		
zma00190	Oxidative phosphorylation	3.79E-04
zma01210	2-Oxocarboxylic acid metabolism	1.47E-03
zma01240	Biosynthesis of cofactors	1.47E-03
zma00020	Citrate cycle (TCA cycle)	1.47E-03
zma00640	Propanoate metabolism	1.47E-03
zma04145	Phagosome	1.47E-03
zma00500	Starch and sucrose metabolism	1.47E-03
zma00280	Valine, leucine and isoleucine degradation	1.93E-03
zma00860	Porphyrin metabolism	7.79E-03
zma01200	Carbon metabolism	8.48E-03
zma00531	Glycosaminoglycan degradation	1.45E-02
zma00620	Pyruvate metabolism	1.45E-02
zma00270	Cysteine and methionine metabolism	1.45E-02
zma00071	Fatty acid degradation	1.45E-02
zma00410	beta-Alanine metabolism	2.32E-02
zma00052	Galactose metabolism	2.74E-02
